# A Non-Contact Fall Detection Method for Bathroom Application Based on MEMS Infrared Sensors

**DOI:** 10.3390/mi14010130

**Published:** 2023-01-03

**Authors:** Chunhua He, Shuibin Liu, Guangxiong Zhong, Heng Wu, Lianglun Cheng, Juze Lin, Qinwen Huang

**Affiliations:** 1 School of Computer, Guangdong University of Technology, Guangzhou 510006, China; 2 Guangdong Provincial People’s Hospital, Guangdong Academy of Medical Sciences, Guangdong Institute of Gerontology, Guangzhou 510080, China; 3 No. 5 Electronics Research Institute of the Ministry of Industry and Information Technology, Guangzhou 510610, China

**Keywords:** image processing, fall detection, feature extraction, MEMS infrared sensor, BP neural network

## Abstract

The ratio of the elderly to the total population around the world is larger than 10%, and about 30% of the elderly are injured by falls each year. Accidental falls, especially bathroom falls, account for a large proportion. Therefore, fall events detection of the elderly is of great importance. In this article, a non-contact fall detector based on a Micro-electromechanical Systems Pyroelectric Infrared (MEMS PIR) sensor and a thermopile IR array sensor is designed to detect bathroom falls. Besides, image processing algorithms with a low pass filter and double boundary scans are put forward in detail. Then, the statistical features of the area, center, duration and temperature are extracted. Finally, a 3-layer BP neural network is adopted to identify the fall events. Taking into account the key factors of ambient temperature, objective, illumination, fall speed, fall state, fall area and fall scene, 640 tests were performed in total, and 5-fold cross validation is adopted. Experimental results demonstrate that the averages of the precision, recall, detection accuracy and *F*_1_*-Score* are measured to be 94.45%, 90.94%, 92.81% and 92.66%, respectively, which indicates that the novel detection method is feasible. Thereby, this IOT detector can be extensively used for household bathroom fall detection and is low-cost and privacy-security guaranteed.

## 1. Introduction

With the development of the economy and the progress of science and technology, the human lifespan continues to extend, and the corresponding issue of population aging has become increasingly prominent, which is a worldwide problem [1]. The population of the elderly is predicted to increase to 1.4 billion by 2030 and 2.1 billion by 2050 [2]. People aging 65 years and older are more vulnerable to fall, and people aged 65 have a risk of 28–35% of falling [3]. The ratio of the elderly to the total population around the world is larger than 10%, which is increasing gradually. According to the World Health Organization (WHO), about 30% of the elderly are injured by falls each year [3], and accidental falls account for a large proportion. Obviously, bathroom falling is one of the most common fall events. Therefore, the capability to detect fall events of the elderly is of great importance since it may cause their long-term stay in hospitals, even death. So far, there are three main fall detection techniques, namely wearable, vision-based, and ambient-based [4,5].

The wearable techniques are mainly based on gyroscopes, accelerometers or an Inertial Measurement Unit (IMU) [6,7,8]. These sensors are embedded in various products, such as belts, watches, necklaces, rings, shoes, bracelets, or wristbands [9,10,11,12,13]. According to the changes of movement characteristics, different fall events can be recognized. Generally, the activity signals are easy to acquire, and the detection accuracies are high. Unfortunately, wearable devices are intrusive-measuring devices since they are attached to the body, causing possible discomfort. Besides, they are power-consuming, and the elders are apt to forget to charge them.

Vision-based techniques are mainly based on video cameras, depth cameras, or thermal cameras [14,15,16,17,18]. They can continuously record the movement images and perform data processing by means of the algorithms of pattern recognition. Once dangerous fall actions are captured, the alarm is triggered. Although they are non-contact detection techniques avoiding potential discomfort, the key limitations are the limited space of application (within the field of view of cameras), high cost, and privacy violation.

The ambient-based techniques are mainly based on pressure sensors, WiFi, or radar sensors [19,20,21]. They can be embedded in the furniture, clothing, floor, and so on. The fall events are identified by the changes in the pressure signals, vital signs signals, or movement signals in specific places. They are unobtrusive and non-invasive; however, they are very expensive, and the installation is also high-cost.

Thereby, aiming at the limitations mentioned above, low-resolution infrared (IR) sensors are adopted to achieve fall detection [22,23]. They have a series of merits, such as low-cost, non-wearable, unobtrusive, non-invasive, and privacy-security guaranteed. Nevertheless, if the number of IR pixels is less and the field of view (FOV) is small, such as 8 by 8 pixels with 60° by 60° FOV, 16 by 4 pixels with 120° by 25° FOV, or 32 by 32 pixels with 33° by 33° FOV, the resolutions and sensitivities will be low [24,25,26,27]. Thus, these IR sensors can be only applied to localized fall detection. To find a balance between privacy security and the image resolution, as well as a compromise between monitoring area and the sensor’s FOV, is very vital. In addition, the motions of the elderly are closely related to fall judgments, whereas it is difficult to detect small motions of the elderly because there is no difference in the thermal image of the low-resolution IR sensor.

Pyroelectric Infrared (PIR) sensors are a useful motion detector [28], which is sensitive to the Infrared Radiation Changes (IRC) induced by human motion. The advantages of PIR sensors are similar to those of thermopile IR sensors, whereas PIR sensors cannot measure the thermogram before or after fall happens. The lack of the human’s contour recognition with the tested thermogram may lead to a misjudgment of fall detection. Therefore, a single sensor, such as a IR or PIR sensor, is hardly competent for fall detection. Recently, multiple fusion sensors, comprising two or more sensors, such as the IR sensor, gyroscope, accelerometer, ElectroCardioGraph (ECG), ultrasonic sensor, depth sensor, or other sensors [29,30,31,32], has been proposed and proven effective to advance the fall detection accuracy.

Given that the consequences of bathroom falls are so severe for the elderly, this paper will focus on studying the fall detection method for bathroom application. According to the analysis mentioned above, this paper will design a novel non-contact fall detector based on the fusion of thermopile IR array sensor and PIR sensor, which is high-accuracy, unobtrusive, non-wearable, non-invasive, low-cost, and privacy-security guaranteed.

As for IR monitoring technique, the fall detection method is mainly composed of three steps [4,5], namely pre-processing, feature extraction, and pattern recognition. Pre-processing is the basis of the whole analysis, which mainly includes filter, position recognition, and contour extraction. However, there are few reports about the related research, especially for position recognition and contour extraction. In order to reduce computing amount and enhance the recognition accuracy, feature extraction is a key step for dimension reduction analysis. Kinematic features (such as contour center, position, velocity, and acceleration, etc.) and Mel-Frequency Cepstral Coefficients (MFCCs) are often extracted as the eigenvectors for fall/non-fall classification [33,34], whereas these feature extraction methods are not useful for fall detection. Finally, some pattern recognition algorithms are applied to accomplish automatic and real-time analysis, among which, support vector machine, Principal Component Analysis (PCA), random forest, fuzzy clustering, and Convolutional Neural Network (CNN) are the prevalent and effective classification algorithms [35,36,37,38,39]. However, some of them are too complex to be realized in the local Microprogrammed Control Unit (MCU).

The mentioned algorithms can be accomplished in the cloud or on the edge [40,41,42]. In view of the requirement that fall detection should be achieved in time and efficiently, edge computing is the best choice, since cloud computing inevitably fails to work once the network connection is unstable. In general, wireless communication and the remote alarm of an IOT device can be achieved by WiFi protocol [4]. That is, a Wi-Fi module can be applied for fall detection [43] or communication, which is a main component of an IOT device. However, considering that the WiFi signal is sometimes unstable and that the device easily drops out of the network, the redundant alarm mechanism can be added to conduct online alarm and positioning in combination with GSM [44,45], which can increase the reliability of life-saving alarms. Owing to the cost, it is also not suitable to transmit the big data by GSM, so edge computing is very essential. The algorithms realized in the MCU are low-cost, low-power-consumption, high-efficiency and high-reliability. However, edge computing in the local MCU requires that the processing algorithms are simple and need few computing resources. Hence, this paper will propose a novel data processing method to satisfy the requirement.

## 2. Materials and Methods

### 2.1. System Design

**Figure 1:**
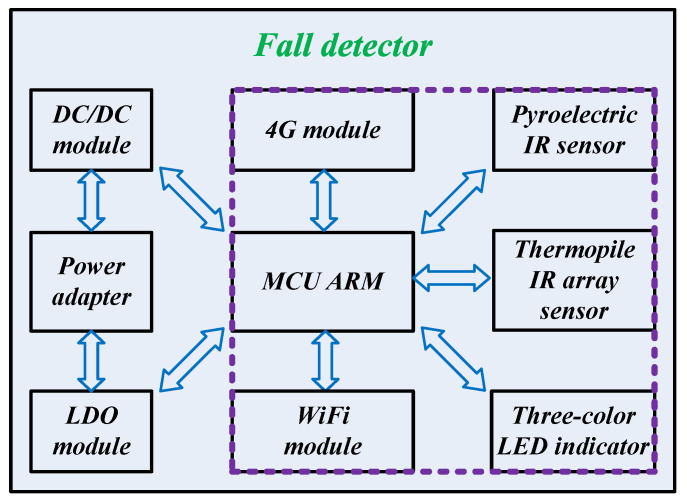
System architecture of an intelligent fall detector.

The system architecture of an intelligent fall detector for bathroom monitoring is depicted in Figure 1, which mainly includes three subsystems, as follows:

(1) Power supply subsystem: Low Dropout Regulator (LDO) and DC/DC converter are powered by a power adapter, then power the whole system.
(2) Processor subsystem: STM32F411 ARM is applied as the edge-computing MCU. WiFi module (WIFI_WRG1, powered by Tuya Co. Ltd., Hangzhou, China) is adopted to conduct remote communication. The alarm information is sent to the management system operated by the caregiver. Meanwhile, the emergency contacts registered in the APP will be contacted with IP call and message. Given that the WiFi signal is sometimes unstable and that the detector is easy to drop out of the network; hence, a 4G module (PAD_ML302, powered by China Mobile Co. Ltd., Chongqing, China) is added in the detector. In this way, the success rate of alarm can be greatly improved through WiFi and 4G dual communication. Furthermore, the positioning with WiFi and 4G modules is also conducive to rapid rescue.
(3) Sensor subsystem: A PIR sensor and a thermopile IR array sensor are applied to detect the body movement and the thermal image, respectively, which are utilized for fall recognition. If a fall event is detected, the detector will send a remote alarm with wireless modules, and the LED indicator will light up in red.

**Figure 2:**
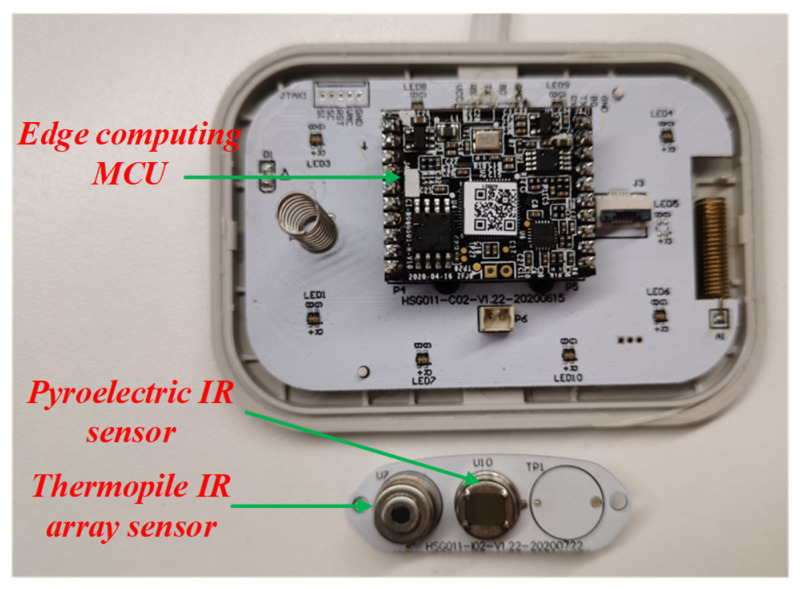
Fall detector based on a MEMS IR sensor and a PIR sensor.

**Figure 3:**
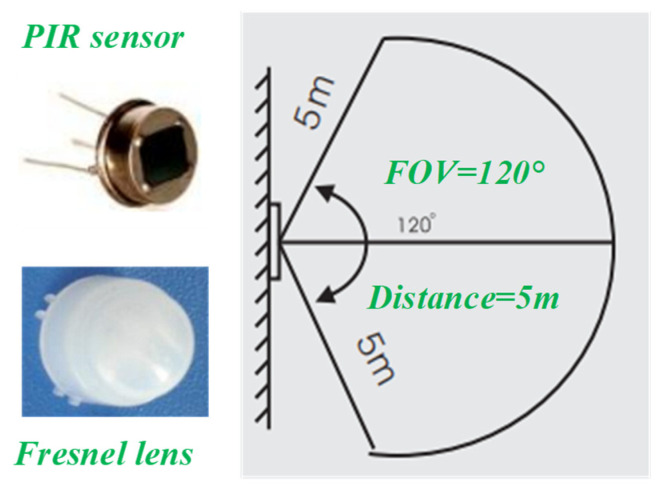
Key parameters of the Fresnel lens and PIR sensor.

The fall detector based on two MEMS IR sensors is shown in Figure 2. It includes an edge-computing MCU, a thermopile IR array sensor, and a PIR sensor. AS312 and 8102-2 are chosen as the PIR sensor and Fresnel lens, respectively, which are made by SENBA Sensing Technology Co., Ltd. (Shenzhen, China). The functions of Fresnel lens are as follows: Firstly, it is used to focus light and filter out light in the non-infrared band. Secondly, the detection area is divided into several bright areas and dark areas, so that the moving objects entering the detection area can generate the change in the thermal infrared signal on the PIR sensor in the form of a temperature change. The detection distance is about 5 m, while the FOV is 120°, as depicted in Figure 3.

**Figure 4:**
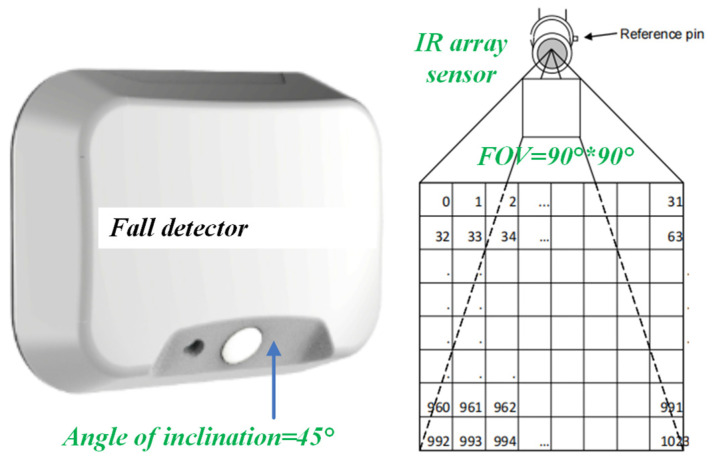
Fall detector is mounted on the sidewall at a height of 1.8 m.

HTPA32x32dR2L2.1 (made by HEIMANN Sensor GmbH, Dresden, Germany) is used as the thermopile IR array sensor. It outputs 32 by 32 pixels of the absolute temperature distribution in a 90° by 90° FOV via I2C bus at a maximum of 5 frames per second. The installed angles of the inclination of the PIR sensor and the IR array sensor are both 45°; thus, the detector can monitor whether there is human activity or a fall in the area below the front, as illustrated in Figure 4. Here, the detector is mounted on the sidewall at a height of 1.8 m, rather than the ceiling. The advantages of this method include: (1) The humidity on the wall being lower than that on the ceiling, so that the reliability is advanced; (2) The detector is easily mounted.

**Figure 5:**
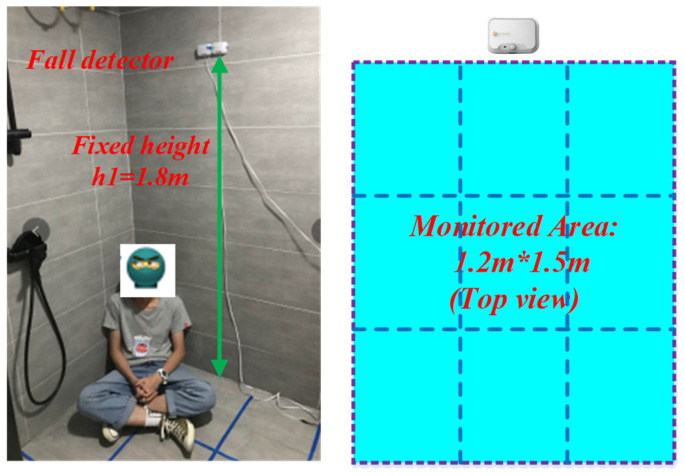
The actual effective monitored area in the bathroom is about 1.2 m by 1.5 m.

In order to improve the moisture-proof performance, the shell of the fall detector is processed by ultrasonic welding, and the sensor opening is waterproof with a silica gel ring, so as to prevent the vapor from seeping into the circuit, advancing the reliability. Now, the detector can achieve IP65 waterproof. Combined with the FOV and inclination angle, the monitored area is calculated to be about 1.8 m by 1.8 m (width by length). Considering the edge effect, the actual effective monitored area is about 1.2 m by 1.5 m, as depicted in Figure 5. In general, the wet area of the bathroom is slippery and someone in that is apt to slip, so the detector should be installed in the wet area of the bathroom. In fact, the area of the wet area is often less than 1.2 m by 1.5 m (i.e., the monitored area).

**Figure 6:**
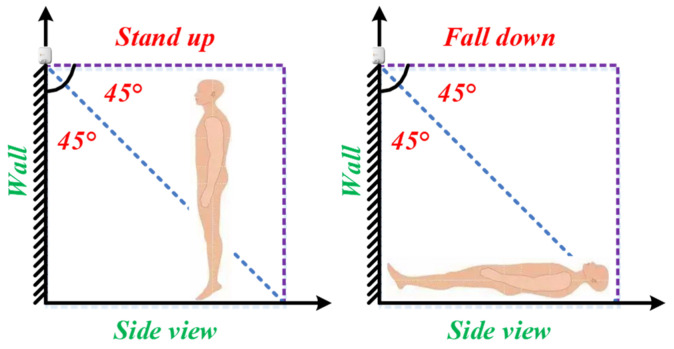
Side view of somebody standing up or falling down.

The side view of someone standing up or falling down is illustrated in Figure 6. It is clear that the human body will appear in the middle and upper part of the thermal image before falling down, whereas the body will appear in the middle and lower part of the thermal image after falling down. That is, the center of the locked body area moves down, and the locked body area will become smaller since the distance increases. Thereby, these changes can be utilized for fall recognition. Besides, combined with a PIR sensor, the detection accuracy can be improved. The fall recognition method consists of the algorithms of image processing, feature extraction, and pattern recognition.

### 2.2. Image Processing

Image processing includes signals filtering and body positioning.

#### 2.2.1. Signal Filtering

**Figure 7:**
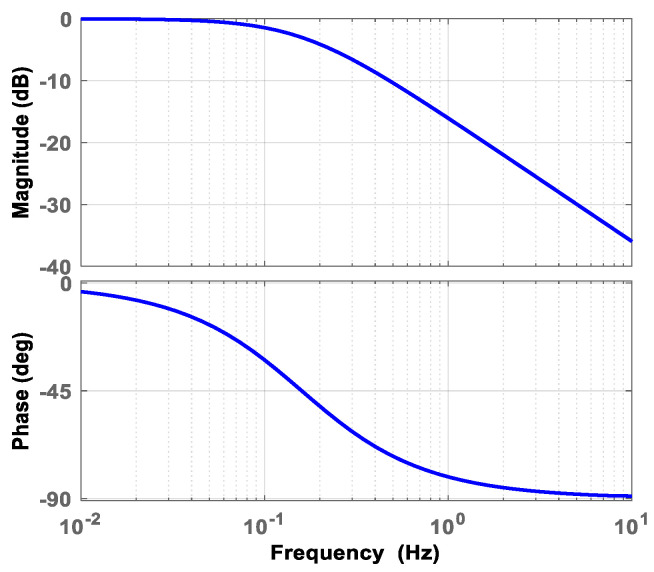
Bode diagram of the first-order low-pass filter.

The thermopile IR array sensor outputs 1024 (=32*32) objective temperature values and 1 ambient temperature value, and the sampling rate *f_s_* is set to 5 Hz. In order to filter the noise, here a first-order Low Pass Filter (LPF) is adopted, whose transfer function in the continuous frequency domain (s domain) is defined as:

(1) $$\frac{Y[s]}{X[s]}=\frac{\omega_{l}}{s+\omega_{l}}$$

where the complex frequency *s* is equal to *jω*, and *ω* is the angular frequency of the signal. *x*(*k*) and *y*(*k*) are the *k-th* input signal and output signal, respectively. *X* and *Y* is the Laplace-transform results of the time domain signals *x* and *y*, respectively. *ω_l_* is the cut-off angular frequency of the LPF, which is set to be 1 since temperature changes slowly. Thus, the cut-off frequency is 0.16 Hz, and the bode diagram of the filter is depicted in Figure 7. After bilinear transformation with (2), the transfer function in the discrete frequency domain (*z* domain) can be derived as (3).

(2) $$s=\frac{2f_{s}(z-1)}{z+1}$$

(3) $$\frac{y[k]}{x[k]}=\frac{\omega_{l}+\omega_{l}z^{-1}}{\omega_{l}+2f_{s}+(\omega_{l}-2f_{s})z^{-1}}$$

**Figure 8:**
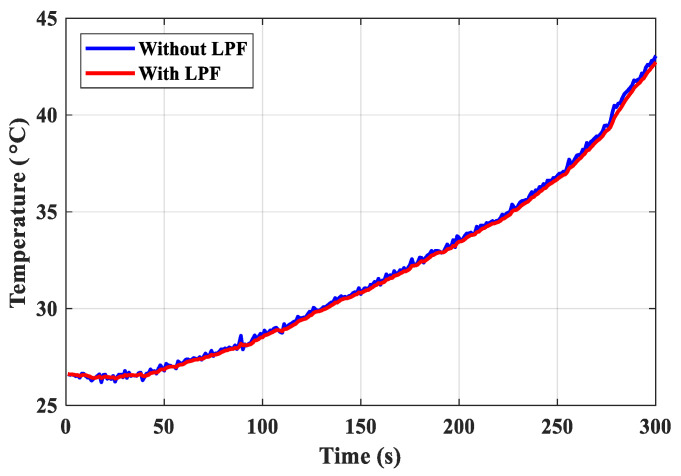
Noise is suppressed by the first-order low-pass filter.

**Figure 9:**
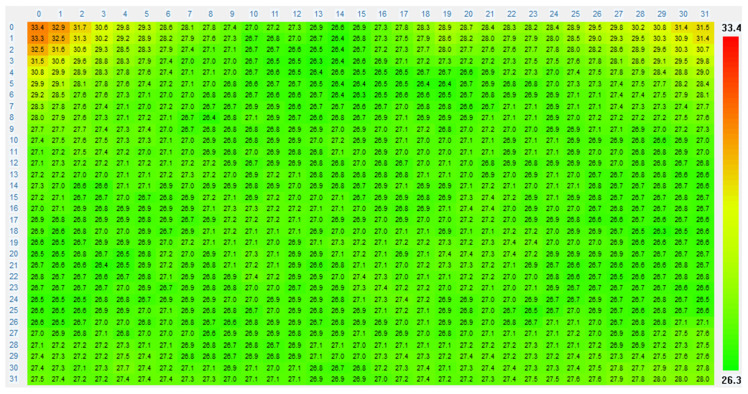
Thermal image of the thermopile MEMS IR array sensor.

Hence, the high-frequency noise can be suppressed by the LPF, as depicted in Figure 8. After filtering, the thermal image is depicted in Figure 9. The background colors green and red represent a low temperature and a high temperature, respectively.

#### 2.2.2. Body Positioning

**Figure 10:**
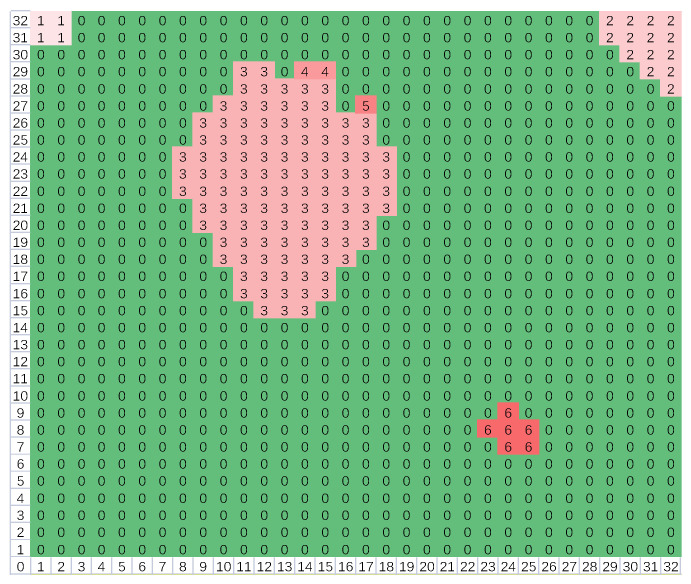
Every point is labeled with a block number after the first boundary scan.

**Figure 11:**
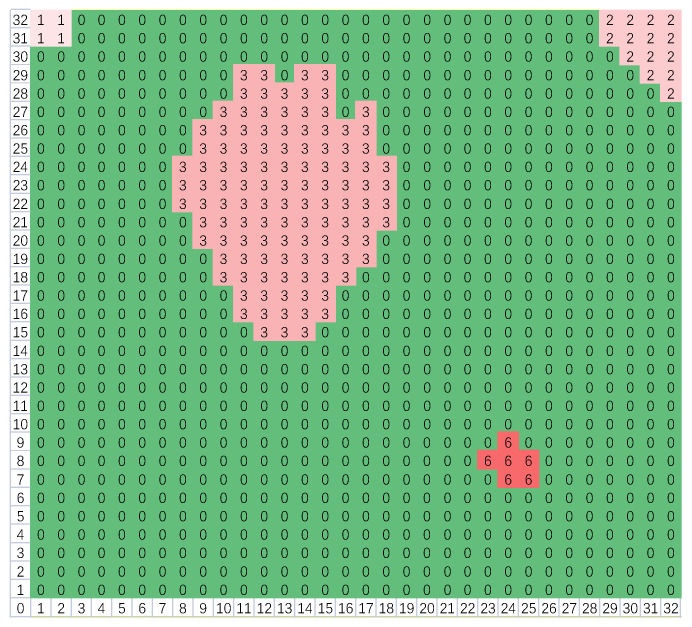
Adjacent blocks are merged after the second boundary scan.

**Figure 12:**
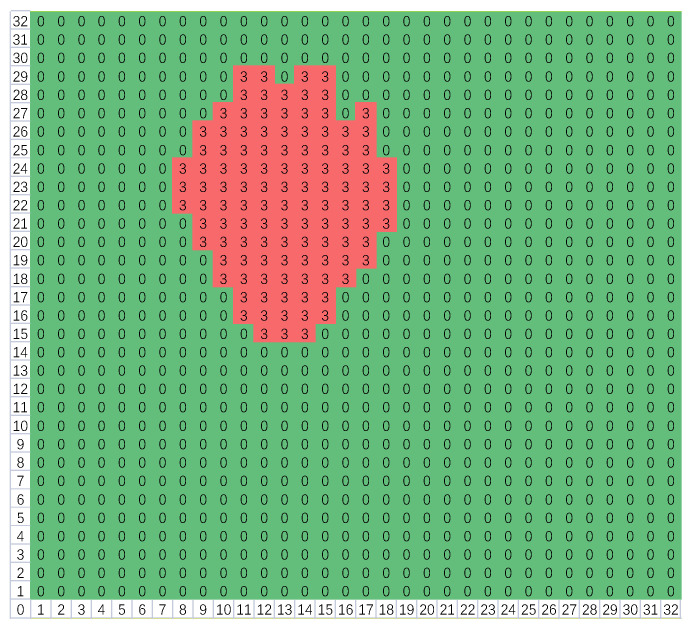
Only the block with the largest area is reserved.

Generally, the background temperature is lower than the human body temperature, so body positioning can be accomplished according to this basis. Meanwhile, some abnormal temperature points higher than 40 °C or lower than 0 °C should be deleted. Then the minimum and maximum of the rest points can be found as *T_min_* and *T_max_*, respectively. Thus, the average of them is set as the threshold *T_th_*. Therefore, temperature points smaller than *T_max_* and larger than *T_th_* can be selected and labeled with a block number bigger than 0. Assume that ***T*** is the current IR temperature array, whose size is 32*32. Here, double boundary scans processing is applied for positioning, which includes three steps:

(1) The first scan: Define a label array ***L*** whose initial values are all 0. Taking boundary extension into account, the size of ***L*** is 34 × 34. Furthermore, set the block number *bn* to be 1. Define a new set ***P*1** as {***L***[*r* − 1][*c* − 1], ***L***[*r* − 1][*c*], ***L***[*r* − 1][*c* + 1], ***L***[*r*][*c* − 1], ***L***[*r*][*c* + 1], ***L***[*r* + 1][*c* − 1], ***L***[*r* + 1][*c*], ***L***[*r* + 1][*c* + 1]}. *r* (1 ≤ *r* ≤ 32) and *c* (1 ≤ *c* ≤ 32) are the row index and column index, respectively. Delete the repeated values or 0 from ***P*1**, then a new set ***P*2** can be obtained. During progressive scanning, if ***P*2** is empty, then *bn* is assigned to ***L***[*r*][*c*], and *bn* is updated as (*bn* + 1). Otherwise, the minimum in ***P*2** will be assigned to ***L***[*r*][*c*]. In addition, if the size of ***P*2** is more than 1, the corresponding blocks are adjacent, then ***P*2** will be added to a relationship table ***Q***. ***Q*** is a two-dimensional (2D) array used to save a series of sets. The pseudo-code is shown as follows:
  (4) $$\begin{array}{l} bn=1;\;i=1;\;Q=\emptyset ;\\ for\;r=1:32\\ for\;c=1:32\\ \left\{\;if\;T[r][c]\in [T_{th},T_{max}\right]\\ \;\{\;if(P2==\emptyset )\\ \;\;\;\{L[r][c]=bn;\;bn=bn+1;\}\\ \;\;\;else\\ \;\;\;\{L[r][c]=min(P2);\\ \;\;\;\;if(size(P2)>1)\;Q[i++]=P2;\}\;\}\\ \} \end{array}$$
  (5) $$\begin{array}{l} for\;i=1:size(Q)\\ for\;j=(i+1):size(Q)\\ \left\{\;if(Q[i]\cap Q[j]\neq \emptyset \right)\\ \;\{Q[i]=Q[i]\cup Q[j];\;Q[j]=\emptyset ;\}\}\\ for\;r=1:32\\ for\;c=1:32\\ \left\{\;for\;i=1:size(Q\right)\\ \;if(L[r][c]\in Q[i])\\ \;\{L[r][c]=min(Q[i]);\\ \;\;\;cnt[min(Q[i])]++;\}\\ \;\} \end{array}$$
(2) The second scan: After the first scan, there may be some adjacent blocks; as depicted in Figure 10, blocks 3, 4, and 5 are connected. Thus, they should be merged together, and the second scan is necessary. Firstly, compare the elements in ***Q*** in pairs; if their intersection is not empty, then merge them to form a union. Secondly, for each element in ***Q***, select the points corresponding to all the block numbers in this set and then modify their labels to the minimum block number of the set. Thus, all the adjacent blocks are merged. As illustrated in Figure 11, blocks 3, 4, and 5 are merged to form block 3. The pseudo-code is depicted as (5), where ***cnt*** is a counter vector applied to record the number of the points of every block.
(3) Owing to the environmental interference, several high-temperature blocks may be picked out. Considering that the area of the human’s block should be the largest, so finally only the largest block is reserved, and others will be all removed. The pseudo-code is shown as (6), where *id* is the block number of the largest block. As depicted in Figure 12, blocks 1, 2, and 6 have been eliminated. If a locked potential body area appears, the signal output by the PIR sensor will be combined together to judge whether there is a fall event, then feature extraction is important.
  (6) $$\begin{array}{l} id=find(max(cnt));\\ for\;r=1:32\\ for\;c=1:32\\ \left\{\;if(L[r][c]\neq id)\;L[r][c]=0;\right\} \end{array}$$

### 2.3. Feature Extraction

A bathroom fall refers to someone slipping in the wet area of the bathroom, and it is related to space and time.

After locking the potential body area, the center coordinate (*X_c_*, *Y_c_*) of the locked area can be calculated by averaging the abscissas and ordinates of all the locked points. Correspondingly, the difference of the center coordinates at adjacent moments (i.e., adjacent frames) is computed as (*dX_c_*, *dY_c_*). Thus, the standard deviation and average of the latest 5 center coordinates (i.e., within 1 s) are (*stdX_c_*, *stdY_c_*) and (*mX_c_*, *mY_c_*), respectively. If *stdX_c_* and *stdY_c_* are both smaller than 1, and the absolute values of *dX_c_* and *dY_c_* are both less than 2, then the locked area is stable, and this moment is named the stable moment. Then (*mX_c_*, *mY_c_*) is assigned to the stable center coordinate (*SX_c_*, *SY_c_*). If the locked area is stable, *flag_sta* is set as 1, otherwise it is set as 0, and (*SX_c_*, *SY_c_*) is not updated, as shown in (7). Similarly, the difference of the stable center coordinates at adjacent stable moments is computed as (*dSX_c_*, *dSY_c_*).

(7) $$\begin{array}{l} if((stdX_{c}<1)\&\&(stdY_{c}<1)\&\&(\left|dX_{c}\right|<2)\&\&(\left|dY_{c}\right|<2))\\ \;\;\;flag\_sta=1;\\ else\;flag\_sta=0; \end{array}$$

**Figure 13:**
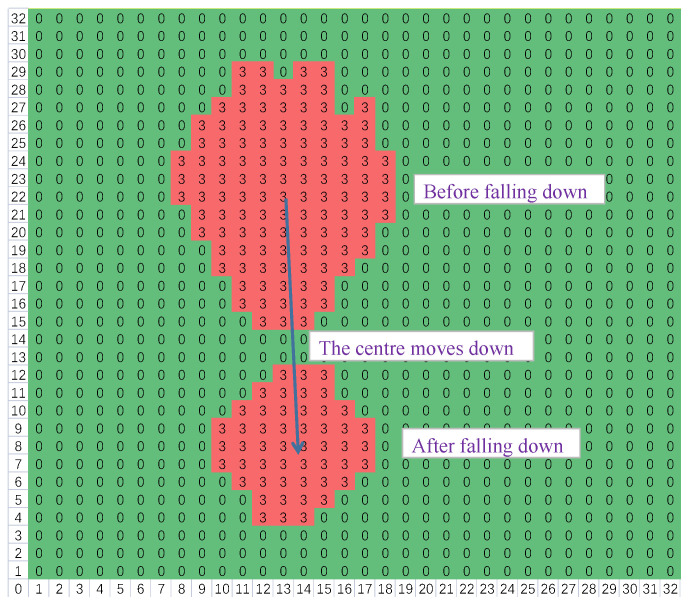
The locked body area moves down, and the number of locked points decreases after falling down.

Assume that ***TSo*** and ***TSn*** are the last and the current IR temperature arrays at adjacent stable moments, respectively, whose sizes are both 32*32. The changes of the temperatures of the new locked area at adjacent stable moments can be estimated by the Euclidean distance (*ED*), as depicted in (8). On the other hand, the mean temperature of the locked area can be calculated as *T_c_*, and the number of the locked points is *N_c_*. Likewise, if the locked body area is stable, *T_c_* and *N_c_* will be updated; otherwise, they remain unchanged. The difference of the mean temperatures of the locked areas at adjacent stable moments is recorded as *dT_c_*, while the ratio of the numbers of the locked points at adjacent stable moments (dividing the latest by the previous) is recorded as *RN_c_*. *SPIR* is the output of the PIR sensor; if human activity appears, *SPIR* is 1, and it will last for 2 more seconds; otherwise, it is 0. If condition (9) is met, there is a recognized fall action, and *flag_act* will be set to be 1.

(8) $$\begin{array}{l} ED=0;\\ for\;r=1:32\\ for\;c=1:32\\ \left\{\;if(L[r][c]\neq 0\right)\\ \;\;ED=ED+(TSn[r][c]-TSo[r][c]{)}^{2};\\ \;\;\}\\ \;\;ED=\sqrt{ED}; \end{array}$$

(9) $$\begin{array}{l} if((\left|dT_{c}\right|<2)\&\&(0.5<RN_{c}<1)\&\&(20<N_{c}<200)\\ \;\&\&(\left|dSX_{c}\right|<LY/3)\&\&(\left|dSY_{c}\right|>LY/2)\&\&(SPIR==1)\\ \;\&\&(1<SX_{c}<30)\&\&(ED>10)\&\&(flag\_sta==1))\\ \;flag\_act=1; \end{array}$$

where *LY* is the last span of the locked area in the *y*-axis direction. Generally, there is a large displacement in the *y*-axis direction after falling, while the displacement in the *x*-axis direction is relatively small. Thus, if someone falls down, the absolute value of *dSX_c_* should be less than *LY*/3, while the absolute value of *dSY_c_* should be larger than *LY*/2. In addition, the difference in the mean temperatures of the locked area before and after falling down should be less than 2 °C. The number of the locked points after falling down should be smaller than that before falling down, but *RN_c_* must be bigger than 0.5, as depicted in Figure 13. Simultaneously, owing to the limited body’s area, *N_c_* should be more than 20 and less than 200. The stable center cannot approximate the boundary, so *SX_c_* should be more than 1 and less than 30. For suppressing the influence induced by residual hot water or other existed heat sources, *ED* should be more than 10. When the new locked area has already existed at the last stable moment, then *ED* must be smaller than 10, and it is not a real fall change.

At the initial moment when *flag_act* becomes 1, the corresponding *T_c_* and *N_c_* are recorded as *T_c_*_0_ and *N_c_*_0_, respectively. Once *flag_act* and *flag_sta* both equal 1, a timer is launched to record the duration *t_d_*, and a PIR sensor is applied to detect human activity. The time of body movement within the latest 1 min is measured to be *t_bm_*. If the condition (10) is satisfied, the fall action disappears, and *flag_act* will be set as 0.

(10) $$\begin{array}{l} if((\left|dX_{c}\right|>2)\left|\right|(\left|dY_{c}\right|>2)\left|\right|(t_{d}>120)\\ \;||(\left|T_{c}-T_{c0}\right|>2)\left|\right|(\left|N_{c}-N_{c0}\right|>N_{c0}/3))\\ \;\;flag\_act=0; \end{array}$$

It means that if the locked center is unstable, if *t_d_* is more than 120 s, if the change of *T_c_* is more than 2 °C, or if the locked area changes by more than 1/3, then *flag_act* should be cleared. Hence, these constraints are conductive to avoid the interference of residual hot water on the ground or other factors.

Generally, if the body movements are more after the thermal image moves down, the monitored objective may squat down to take a bath or try to get up after falling down, thus no alarm is required in these cases. Only if the locked area is basically stable after moving down, and body movements are fewer and fewer, can it be identified as a fall. Due to the body movements detected by a PIR sensor, some abnormal interference resulting from light, hot water, and sunlight can be eliminated effectively.

In addition, the standard deviations of *N_c_* and *T_c_* within 1 s are calculated as *stdN_c_* and *stdT_c_*, respectively. Then *stdX_c_*, *stdY_c_*, *t_d_*, *t_bm_*, *stdN_c_*, *stdT_c_*, *flag_sta* and *flag_act* will be applied to accomplish pattern recognition. To sum up, the risk of misjudgment can be reduced by a series of constraints. Considering that some key parameters are extracted based on the data in the past one minute, so the response time of the detection system is about one minute. Here, the response time of the real-time detection system is hard to advance in order to avoid misjudgment.

### 2.4. Pattern Recognition

**Figure 14:**
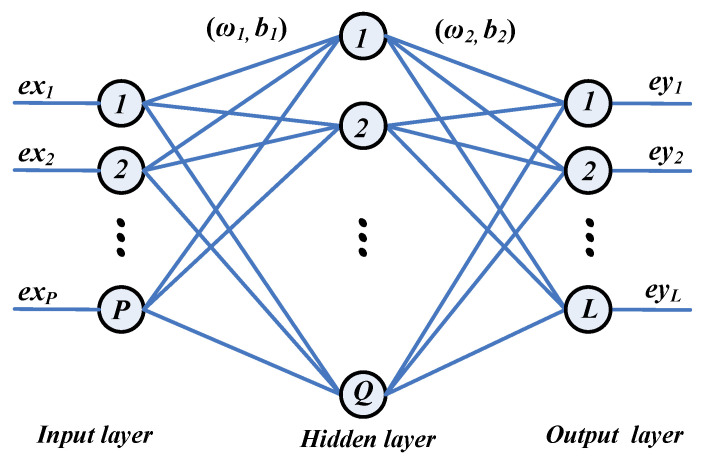
Architecture of a simple BP neural network system.

A BP (back propagation) neural network has strong abilities of generalization and nonlinear mapping, so it can be widely used in the learning, prediction, and identification of nonlinear systems. In this paper, a three-layer BP neural network is adopted for fall recognition, as illustrated in Figure 14. ***EX*** is the input matrix of the input layer; ***EZ*** is the output matrix of the hidden layer, and ***EY*** is the output matrix of the output layer. In addition, tansig is the transfer function of the hidden layer, while relu is the transfer function of the output layer. Trainlm is selected as the network’s training function, and mean square error is utilized for performance evaluation. The weights matrices ***ω*_1_**, ***ω*_2_** and the threshold matrices ***b*_1_**, ***b*_2_** are adjusted with the steepest descent method until the training error (*ERR*) reaches the setting target, as shown in (11).

(11) $$ERR=(EY-Tg{)}^{\prime }\times (EY-Tg)/2$$

where ***Tg*** is the expected target. After training, ***ω*_1_**, ***ω*_2_**, ***b*_1_**, ***b*_2_** are confirmed, and the network can be applied to accomplish the fall recognition.

As mentioned above, the input matrix is [*stdX_c_*, *stdY_c_*, *t_d_*, *t_bm_*, *stdN_c_*, *stdT_c_*, *flag_sta*, *flag_act*], and the output matrix is [non-fall or fall]. Hence, the number of the neurons in the input layer is 8, and the number of the neurons in the output layer is 1. In order to advance the learning effect, the number of neurons in the hidden layer is set to 20. Hence, this BP neural network is very simply realized in the MCU.

## 3. Experimental Results

### 3.1. Performance Indices

Based on the test platform illustrated in Section 2 and the detection method described in Section 3, a series of experiments can be performed. Here, recall (*RE*), precision (*PR*), detection accuracy (*ACC*), and *F*_1_*-Score* are four important performance indices used to evaluate the recognition performance, as defined in (12).

(12) $$\begin{array}{l} RE=\frac{TP}{TP+FN},\;PR=\frac{TP}{TP+FP}\\ ACC=\frac{TP+TN}{TP+TN+FP+FN}\\ F_{1}\text{-}Score=\frac{2\times RE\times PR}{RE+PR} \end{array}$$

where *TP* is the number of the fall events detected correctly, *FN* is the number of the fall events detected incorrectly, *TN* is the number of the non-fall events detected correctly, and *FP* is the number of the non-fall events detected incorrectly.

### 3.2. Test Scheme

The key factors for bathroom fall detection are listed in Table 1. Considering that ambient temperature has the greatest impact on the IR detection effect, five common room temperature points (such as 18 °C, 21 °C, 24 °C, 27 °C, 30 °C) can be selected to perform the experimental tests. It can be controlled by a bathroom heater or central air conditioner. The tests are accomplished in the bathroom depicted in Figure 5. In this work, a central air conditioner was finally adopted to control the ambient temperature. The controller and temperature sensor of the central air conditioner were placed in the living room, so the controlled ambient temperature was actually the temperature of the living room. If the bathroom has no running hot water, the temperature in the bathroom is basically the same as that in the living room. If the hot water is running, the temperature in the bathroom will actually be slightly higher than the ambient temperature in the living room. Thus, in this way, the actual ambient temperature can be simulated.

Furthermore, the other factors have only two levels to reduce the test amount. For the tested objectives, here a young woman with height of 1.6 m and a young man with height of 1.8 m were recruited to simulate the bathroom falls of the elderly. They have signed an informed consent form and a privacy protection agreement. For estimating the influence of illumination, LED light and sunlight are taken into account. For estimating the response speed, fast fall and slow fall are selected. Given that the state after falling down is also important, two cases of sitting and lying on the ground should be considered. In addition, falling at the boundary or in the center are the two common cases. In general, the bathroom fall may happen when taking a shower or not taking a shower. For each combination of factors, falls and non-falls should be considered; thus, the test amount is 640 (=128 × 5). For each test, nine data composed of eight input parameters and one output are obtained.

### 3.3. Test Results

Considering that the test cases are limited, five-fold cross validation is adopted. Based on five different ambient temperatures, the test data are separated into five sets (i.e., S1, S2, S3, S4, S5), and every set has 128 tests. Here, S1 is the data set corresponding to 18 °C, while S5 is the data set corresponding to 30 °C. Thus, for every fold, one data set is utilized for validation, and the other four data sets are used for training. Taking Fold No. 5 as an example, S5 is utilized for validation, and {S1, S2, S3, S4} are used for training.

**Figure 15:**
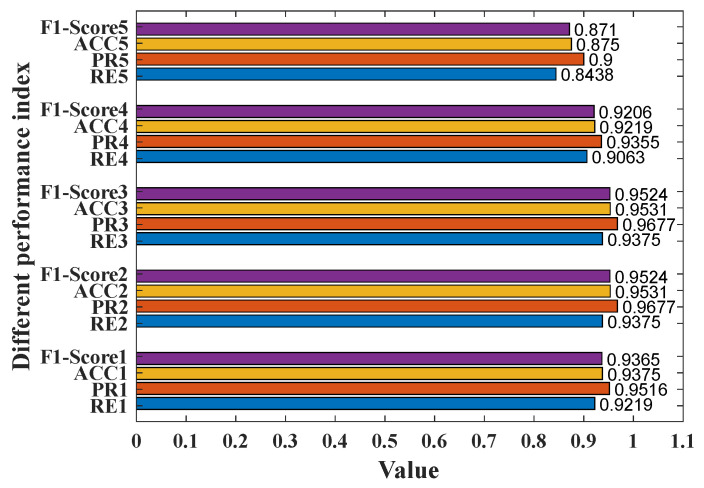
Different performance indices of 5-fold cross validation.

For every fold, the *TP*, *FN*, *TN*, *FP* of the validation set are calculated and shown in Table 2, then the *PR*, *RE*, *ACC*,and *F*_1_*-Score* can be obtained, as shown in Figure 15. The averages of *TP*, *FN*, *TN*, and *FP* are 58.2, 5.8, 60.6 and 3.4, respectively. Besides, the averages of *PR*, *RE*, *ACC,* and *F*_1_*-Score* are measured to be 94.45%, 90.94%, 92.81%, and 92.66%, respectively. These results indicate that the misjudgment of non-falls is less than that of falls.

In addition, *ACCs* of Fold No. 2 and Fold No. 3 are both 95.31%, and the corresponding ambient temperatures are 21 °C and 24 °C. The ambient temperatures of Fold No. 4 and Fold No. 5 are 27 °C and 30 °C, and the *ACCs* of them are 92.19% and 87.5%, respectively. Although the ambient temperature of Fold No. 1 is the smallest (i.e., 18 °C), the training data set are {S2, S3, S4, S5}, which is worse than the training data sets of Fold No. 2 and Fold No. 3, that is why *ACC* of Fold No. 1 is 93.75%, less than those of Fold No. 2 and Fold No. 3. To sum up, for IR sensor application, high ambient temperature will affect fall detection accuracy.

## 4. Discussion

It is obvious that *FN* is still a little big and that this performance is not enough. Considering that the ambient temperature has a great impact on *ACC*, if we want to improve it, maybe more fusion sensors should be adopted. For instance, a voice alarm module can be added. MEMS microphone could be applied to acquire the ambient noise and human’s voice, then voice recognition could be performed to judge whether there was a fall event or voice alarm. These will be our next work.

A comparison of different fall detection methods is summarized and listed in Table 3. It indicates that the accuracies of wearable techniques and vision-based techniques are the best, reaching from 96% to 100%. However, wearable techniques are not easily accepted by the elderly, and vision-based techniques have high-cost and constitute a privacy violation. The accuracies of ambient-based techniques are the worst, and they are expensive. The accuracies of low-resolution IR sensors and multi-sensors are similar to that of this work, but those methods have not taken complex bathroom application scenes into consideration. Hence, this work is very significant.

## 5. Conclusions

In this article, a non-contact fall detector based on a MEMS PIR sensor and a thermopile IR array sensor was designed to detect bathroom falls. Besides, image processing algorithms with a low-pass filter and double boundary scans were put forward in detail. Then, the statistical features of the area, center, duration, and temperature are extracted. Finally, a three-layer BP neural network was adopted to identify the fall events. Taking into account the key factors of ambient temperature, objective, illumination, fall speed, fall state, fall area, and fall scene, in total 640 tests were performed, and five-fold cross validation was adopted. Experimental results demonstrate that the averages of the precision, recall, detection accuracy, and *F*_1_*-Score* were measured to be 94.45%, 90.94%, 92.81% and 92.66%, respectively, which indicates that the novel detection method is feasible. In addition, for IR sensor application, the fall detection accuracy decreases as the ambient temperature increases.

Hence, this IOT detector can be extensively used for household bathroom fall detection, which is low-cost and privacy-security guaranteed. Given that the detection accuracy is not high enough, more fusion sensors and more tests should be adopted, and these will be our next work.

## Figures and Tables

**Table 1:** Key factors for bathroom fall tests.

Factor	Level
Ambient temperature	18 °C, 21 °C, 24 °C, 27 °C, 30 °C
Objective	female (1.6 m), male (1.8 m)
Illumination	LED light, sunlight
Fall speed	fast, slow
Fall state	sitting, lying
Fall area	at the boundary, in the center
Fall scene	shower, without shower

**Table 2:** Test results of bathroom fall experiments.

Fold No.	*TP*	*FN*	*TN*	*FP*
1	59	5	61	3
2	60	4	62	2
3	60	4	62	2
4	58	6	60	4
5	54	10	58	6
Average	58.2	5.8	60.6	3.4

**Table 3:** Comparison of different fall detection methods.

Detection Method	Sensor	Accuracy	Comment	References
Wearable techniques	inertial sensors, IMU	96~100%	The elderly are not willing to wear the product and are apt to forget to charge it.	[6,7,8,9,10,11,12,13]
Vision-based techniques	video cameras, depth cameras, or thermal cameras	96~100%	high-cost and privacy violation	[14,15,16,17,18]
Ambient-based techniques	pressure sensors, WiFi, or radar sensors	85~90%	expensive, and the accuracy is not high	[19,20,21]
IR sensors	low resolution IR sensors	85~97%	Complex bathroom application scenes are not considered.	[22,23,24,25,26,27]
Multi-sensors	gyroscope, accelerometer, ECG, ultrasonic sensor, depth sensor, etc.	90~97%	Complex bathroom application scenes are not considered.	[29,30,31,32]
This work	PIR + low resolution IR sensor	87.5~95.31%	suitable for bathroom application	/

## Data Availability

Not applicable.
